# Circadian Rhythms in Exudative Age-Related Macular Degeneration: The Key Role of the Canonical WNT/β-Catenin Pathway

**DOI:** 10.3390/ijms21030820

**Published:** 2020-01-27

**Authors:** Alexandre Vallée, Yves Lecarpentier, Rodolphe Vallée, Rémy Guillevin, Jean-Noël Vallée

**Affiliations:** 1 DACTIM-MIS, Laboratory of Mathematics and Applications (LMA), UMR CNRS 7348, University of Poitiers, CHU de Poitiers, 86021 Poitiers, France; remy.guillevin@chu-poitiers.fr; 2 Centre de Recherche Clinique, Grand Hôpital de l’Est Francilien (GHEF), 77100 Meaux, France; yves.c.lecarpentier@gmail.com; 3 University Hospital Group of Paris-Seine-Saint-Denis, APHP, University of Paris-13 Sorbonne Paris-Cité, 93000 Paris, France; rod.vallee@gmail.com; 4 CHU Amiens Picardie, University of Picardie Jules Verne (UPJV), 80000 Amiens, France; valleejn@gmail.com; 5 Laboratory of Mathematics and Applications (LMA), UMR CNRS 7348, University of Poitiers, 86021 Poitiers, France

**Keywords:** exudative AMD, circadian rhythms, WNT/β-catenin pathway, aerobic glycolysis, Warburg effect

## Abstract

Age-related macular degeneration (AMD) is considered as the main worldwide cause of blindness in elderly adults. Exudative AMD type represents 10 to 15% of macular degeneration cases, but is the main cause of vision loss and blindness. Circadian rhythm changes are associated with aging and could further accelerate it. However, the link between circadian rhythms and exudative AMD is not fully understood. Some evidence suggests that dysregulation of circadian functions could be manifestations of diseases or could be risk factors for the development of disease in elderly adults. Biological rhythms are complex systems interacting with the environment and control several physiological pathways. Recent findings have shown that the dysregulation of circadian rhythms is correlated with exudative AMD. One of the main pathways involved in exudative AMD is the canonical WNT/β-catenin pathway. Circadian clocks have a main role in some tissues by driving the circadian expression of genes involved in physiological and metabolic functions. In exudative AMD, the increase of the canonical WNT/β-catenin pathway is enhanced by the dysregulation of circadian rhythms. Exudative AMD progression is associated with major metabolic reprogramming, initiated by aberrant WNT/β-catenin pathway, of aerobic glycolysis. This review focuses on the interest of circadian rhythm dysregulation in exudative AMD through the aberrant upregulation of the canonical WNT/β-catenin pathway.

## 1. Introduction

Age-related macular degeneration (AMD) is considered as the main worldwide cause of blindness in elderly adults [1]. AMD progression is initially characterized by the primary influence of debris accumulation in the early step, whereas the late step presents an accumulation of retinal epithelial dysfunctions. AMD is divided into two types: “non-exudative” and “exudative” AMD. Non-exudative AMD is marked by a progressive loss of the retinal pigment epithelium (RPE) cell layer and thinning of the retina, while exudative AMD is characterized by choroidal neovascularization (CNV) and sub-retinal neovascular fibrous tissue [2], leading to central vision deterioration [3]. CNV is defined by abnormal blood vessels from the choroid underneath the macula [4]. The exudative AMD type represents 10 to 15% of macular degeneration cases, but this is the main cause of vision loss and blindness [5,6] because CNV participates in 80% of this vision loss due to AMD [7]. CNV initiation is correlated with the enhancement of the proangiogenic factor vascular endothelial growth factor (VEGF) [8]. Currently, the molecular pathways involved in AMD still remain to be clarified. Nevertheless, the aging process is a main risk factor for neurodegeneration and then for exudative AMD. This process can disturb molecular pathways involving homeostatic mechanisms [9]. Exudative AMD undergoes metabolic reprogramming, closely associated with aging, called aerobic glycolysis, or the Warburg effect [10].

Circadian rhythm (CR) changes are associated with aging and could further accelerate it [11]. However, the link between CRs and exudative AMD is not fully understood. The dysregulation of circadian functions could be manifestations or risk factors for the development of diseases in elderly adults [12,13,14,15]. Indeed, circadian clocks have a main role in physiological and metabolic functions [16], and one of the key integrators of these metabolic mechanisms is the canonical WNT/β-catenin pathway [17,18]. Exudative AMD is associated with the upregulation of this WNT/β-catenin pathway [19], leading to the activation of aerobic glycolysis [20]. In parallel, the dysregulation of CRs upregulates the WNT/β-catenin pathway [21], which in turn participates in AMD. This review focuses on the interest of CR dysregulation in exudative AMD through the aberrant upregulation of the canonical WNT/β-catenin pathway.

## 2. Circadian rhythms (CRs)

The endogenous characteristic of CRs is an innate oscillation associated with a period of over one day. All of the studied organisms show this oscillatory process. Numerous cell functions present temporal variations driven by these oscillatory and circadian ways including gene expression, metabolic reprogramming, and molecular and cellular pathways. Different integration levels allow for the study of CRs as endocrinal, physiological, and neuronal cell behaviors. Although the coordination and the modulation of CRs are organized by specific pacemaker structures, the primary circadian oscillations are controlled at the cell level. These oscillations are determined by numerous clock genes [22]. The control of the circadian clock is based on an intracellular temporal tracking system that allows anterior organisms to change direction and thus adapt their behavior and the physiology of their life span [23]. It is well known that in many animal species, the circadian clock is formed by a specific set of transcription factors that constitutes its molecular architecture. These factors are used in a double feedback modulated by a cell-autonomous manner [24].

Endogenous oscillations generate a freewheeling period, which is close to 24 h, at constant ambient conditions to maintain the organism. These oscillators, at the molecular level, are based on the products of clock regulator genes organized in a transcriptional feedback loop. Circadian oscillations are the product of post-transcriptional modifications of proteins [25]. A complex loop operates with clock gene transcriptional activators and in turn, the clock genes act with a negative feedback role to inhibit their own expression by disrupting the activity of their activators [26]. Several input pathways involve environmental information, which interact with the different compounds of the oscillators. The oscillators are synchronized with the 24 h solar day. The input pathways generate a day-time to transpose it by the oscillators to the output pathways. These output pathways control and regulate the expression of circadian clock genes to generate the rhythmicity.

Moreover, the output pathways are predicted to be rhythmic and then controlled by the clock gene transcription factors. These compounds, in turn, regulate downstream the circadian clock genes in a time-of-day-specific manner [27]. This system can synchronize with its environmental time through its internal clock. To respect the environment, the input pathways are vital to maintain this timing for oscillators. The process, named entrainment, acts on the input pathways to reset the activity of the oscillators and stay in a conformed 24 h period of the environment [27]. Environmental cues can be detected by input pathways, which in turn can modulate several mechanisms to control the activity or level of compounds of oscillators to keep a correct time of day expression. This phenomenon is observed in several environmental cues including nutrition, social interactions, and temperature [28,29]. Furthermore, the clock allows a strategy, named gating, to restrict responses to environmental cues at specific day times. Diurnal mammals are insensitive to a light pulse during the day. Nevertheless, during the night, a light pulse can advance or delay the clock to synchronize diurnal mammals with the environment [24]. Environmental signals can interact with molecular oscillators in some cells in complex multicellular organisms. In unicellular organisms, each cell is modulated by oscillators in response to light [30]. However, in multicellular organisms, only a part of the cells has sensory capabilities leading to clock oscillators. The oscillators, and thus, the overall rhythmicity of organisms, are concentrated into compounds including a master pacemaker and peripheral oscillators [31]. Faced with these sensory inputs, the organism presents some nervous systems that possess environmental cue abilities as central oscillators or pacemakers, rather than individual cells. In humans, sensory clock inputs are localized in the brain, where signals from the master pacemaker lead to oscillators in some tissues of the organism.

Photo-entrainment of the suprachiasmatic nucleus processes through pathways from a subpopulation of retinal ganglion cells (RGCs), which are melanospin-expressing and intrinsically photosensitive (ipRGCs) [32]. These retinal ganglion cells receive and perceive the light, and transmit this information to the master pacemaker (localized in the hypothalamus) by neural connections [33]. The central pacemaker synchronizes the oscillators to other tissues using circadian input pathways from the nervous system to peripheral cell systems. The central system allows cellular oscillations within tissues in an adequate phase to maintain the resonance between the different cellular rhythms involved by the environment [34]. Melatonin operates as a major synchronizer in humans and provides temporal feedback to the oscillators within the nervous system for controlling the circadian phase and the rhythm stability [35]. In humans, as in other mammals, melatonin is considered as an important influencer of CRs through its action on receptors in the nervous system [36]. 

## 3. Circadian Clock 

**Figure 1:**
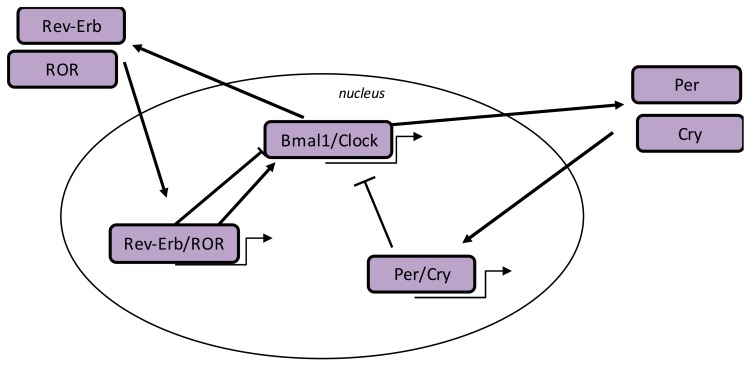
Circadian clock genes. The clock process is a stimulatory circle involving the *Bmal1*/Clock heterodimer that activates the transcription of *Per* and *Cry* genes, and the inhibitory feedback circle with the *Per/Cry* heterodimer that translocates to the nucleus and represses the transcription of the *Clock* and *Bmal1* genes. An additional circle implicates the *RORs* and *Rev-Erb* factors with positive feedback by *RORs* and negative feedback by *Rev-Erb*. Abbreviations: circadian locomotor output cycles kaput (*Clock*), brain and muscle aryl-hydrocarbon receptor nuclear translocator-like 1 (*Bmal1*), period (*Per*), cryptochrome (*Cry*), and retinoid-related orphan receptors (*RORs*).

In humans, many biological mechanisms are modulated by the circadian “clock” (circadian locomotors output cycles kaput) (Figure 1). The circadian clock is localized in the hypothalamic suprachiasmatic nucleus (SCN). CRs are endogenous and entrainable free-running 24 h periods. Numerous transcription factors can act on CRs. These factors are called circadian locomotor output cycles kaput (*Clock*), brain and muscle aryl-hydrocarbon receptor nuclear translocator-like 1 (*Bmal1*), Period 1 (*Per1*), Period 2 (*Per2*), Period 3 (*Per3*), and Cryptochrome (*Cry 1* and *Cry 2*) [37,38]. These factors are controlled by positive and negative self-loop-regulation modulated by CRs [24,39]. *Clock* and *Bmal1* heterodimerize, leading to the transcription of *Per1, Per2, Cry1*, and *Cry2* [40]. The *Per/Cry* heterodimer downregulates its stimulation through a negative feedback. This heterodimer translocates back to the nucleus to directly inhibit the *Clock/Bmal1* complex and then downregulate its transcription [40]. The *Clock/Bmal1* complex stimulates the transcription of retinoic acid-related orphan nuclear receptors, *Rev-Erbs*, and retinoid-related orphan receptors (*RORs*). Through a positive feedback loop, *RORs* activate the transcription of *Bmal1*, whereas through a negative feedback loop, *Rev-Erb* downregulates their transcription [40]. 

## 4. Circadian Clocks in Exudative Age-Related Macular Degeneration 

**Figure 2:**
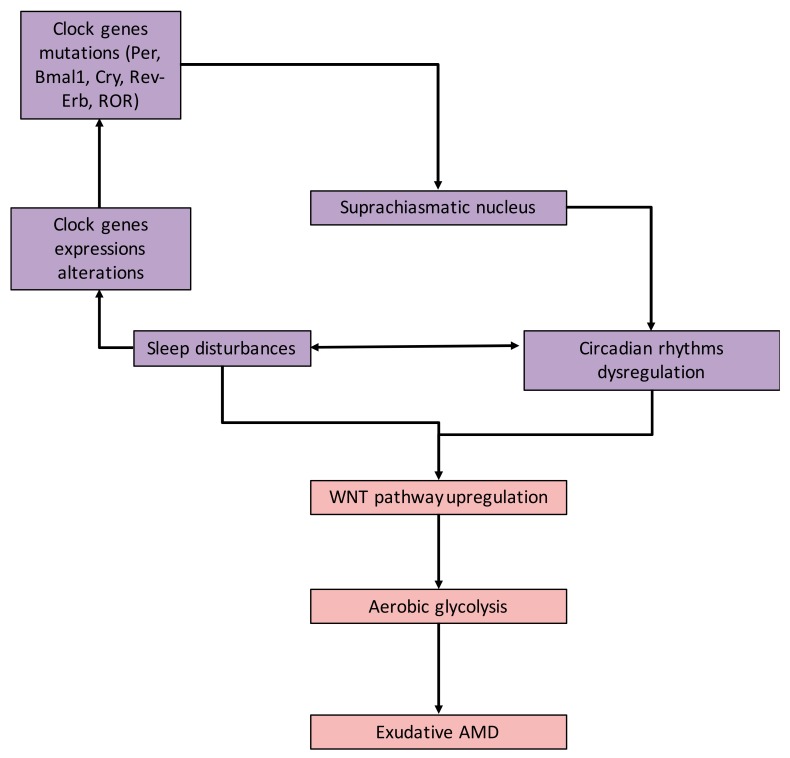
Circadian rhythms (CRs) and exudative Age-related macular degeneration (AMD). Relationship between exudative AMD, CRs, and sleep disturbance. Alterations in clock genes contribute to the dysregulation of circadian sleep rhythmicity. CR deregulation leads to brain metabolism alterations (i.e., aerobic glycolysis), contributing to exudative AMD. Abbreviations: age-related macular degeneration (*AMD*), circadian locomotor output cycles kaput (*Clock*), brain and muscle aryl-hydrocarbon receptor nuclear translocator-like 1 (*Bmal1*), period (*Per*), cryptochrome (*Cry*) and retinoid-related orphan receptors (*RORs*).

A complex mechanism is involved in the retinal circadian system (Figure 2). This mechanism is composed of a complex circadian system associated with the generation of numerous CRs. Currently, the interaction between AMD and CRs has been poorly studied. However, some evidence has highlighted that physiological ocular mechanisms are controlled by CRs in humans. Photoreceptors renew their light-sensitive outer segments through disk shedding and the subsequent formation of new disks from the cilium of the inner segment. In vertebrates, CRs participate in the synchronization of outer segment renewal [41,42]. This phenomenon occurs once per day. Moreover, light onset is associated in synchronization with rod shedding in animals [43,44]. The outer segments should be shed, and the formation of new outer segments operates in coordination to maintain a constant length of photoreceptors. Some experimental studies in animal models have highlighted this association between CRs and AMD [45]. Dysregulation of the circadian clock by constant light exposure in zebrafish enhances the process of angiogenesis [45]. Activation of *Bmal1* and *Per2* leads to vascular initiation. *Bmal1* targets *VEGF* in its promoter region to enhance its activity. In parallel, the deletion of the promoter region of the *VEGF* gene is associated with the inhibition of this promoter. Thus, these results could extend the development of angiogenesis in humans as the same process is observed in animals [46]. Dysfunction of the circadian clock system does not interact only with one physiological phenomenon, but participates in the progression of some diseases [47]. Melatonin is daily rhythmically produced by photoreceptors in the retina with an acrophase at night [48]. Numerous studies have shown that melatonin is involved in AMD progression [49,50]. Even if few studies have focused on the link between CRs, melatonin production, and AMD, some studies suggest that the melatonin rhythm is reversed in AMD [51]. 

## 5. Aerobic Glycolysis and AMD

In mammalian cells, glucose is the main source of energy. All tissues require ATP to operate in physiological conditions. Cells produce ATP through the drop-in oxidation state from glucose (energy-rich molecule) by cell respiration, down to producing CO_2_ at the end. This mechanism operates in an aerobic or anaerobic manner, which depends on the available O_2_. Glycolysis, which occurs in the cytoplasm of cells, is the first stage in the glucose metabolism pathway. The presence of O_2_ is major because glucose oxidation under aerobic conditions leads to 32 molecules of ATP per mol of glucose. Under anaerobic conditions, only two molecules of ATP can be produced. Aerobic glycolysis operates in two steps. The first step occurs in the cytosol and involves the conversion of one glucose into two molecules of pyruvate, resulting in NADH production and generating two molecules of ATP. In normal conditions, when oxygen is available, the energy contained in NADH is further released via re-oxidization of the mitochondrial chain and leads to the release of 38 molecules of ATP per molecule of glucose. Under aerobic glycolysis, this NADH, instead of re-oxidization, converts pyruvate to lactate [52]. Thus, glucose is metabolized in order to produce ATP through cytosolic glycolysis. Glucose entry into the tricarboxylic acid (TCA) cycle is modulated by pyruvate dehydrogenase complex (*PDH*) [53]. In normal conditions, pyruvate is oxidized to acetyl-coA in mitochondria by the *PDH*. Acetyl-coA then translocates to the TCA cycle for oxidation. Under aerobic glycolysis, pyruvate is converted into lactate in the cytosol. This phenomenon is called aerobic glycolysis or the Warburg effect.

Several and multifactorial conditions could be associated with the pathogenesis of exudative AMD, including UV light exposure [54] and aging [55]. Nevertheless, few studies have highlighted that aerobic glycolysis can participate in the promotion of exudative AMD [56]. Photoreceptors from retinal glial cells produce lactate by glycolysis in the normal retina (Müller cells). Glucose, and then lactate, is consumed for oxidative metabolism in the photoreceptors [57]. Müller cells mainly produce ATP through the aerobic glycolysis process and less through oxygen consumption [58]. The retina needs a continuous flow in oxygen and glucose to maintain its physiological functions. Some studies have shown that the retina can require aerobic glycolysis as well as cancer cells [59,60,61,62,63]. To initiate the vision process, the retina can also utilize oxidative phosphorylation and aerobic glycolysis [62]. Nevertheless, the majority of glucose consumed by isolated retina, more than 80%, is produced by aerobic glycolysis [63]. The observed production of lactate in the retina is associated with a high level of O_2_ consumption [5,6,64]. 

The activated molecular pathways involved in aerobic glycolysis [20,65,66] have also been observed in exudative AMD. The PI3K/Akt pathway is over-activated in exudative AMD [67,68,69] and is associated with the stimulation of *HIF-1α* and *VEGF* activities [69]. Activated *HIF-1α* releases VEGF that affects the functions of choroid and retinal endothelial cells and initiates the angiogenesis process in exudative AMD [70,71,72]. Pyruvate kinase activity produced by photoreceptors is associated with the involvement of aerobic glycolysis [73]. *PKM2* is over-stimulated in exudative AMD [74]. In AMD, activation of *EGFR* leads to the transactivation of β-catenin and the transcription of *cyclin D1* by a *PKM2* positive feedback [74,75]. 

Recent findings have shown that lactate levels are increased and pyruvate levels are decreased in exudative AMD. This suggests that aerobic glycolysis is preferred over oxidative phosphorylation in exudative AMD molecular process [76]. These results have shown a possible stimulation of *LDH-A* leading to a production of lactate and a decrease in the pyruvate level entering the TCA cycle [76]. Moreover, in retina cells, the photoreceptors can metabolize glucose through the process of aerobic glycolysis in order to protect them against oxidative damage [77].

## 6. Neovascularization and Warburg Effect

CNV initiation involves the stimulation of *VEGF* activated by the WNT/β-catenin pathway [78,79]. The decrease of *DKK1*, a WNT inhibitor, is associated with exudative AMD, and then with the severity of CNV [80]. In exudative AMD, VEGF expression is stimulated by the aberrant WNT/β-catenin pathway [78,81,82]. The WNT/β-catenin pathway can directly stimulate the expression of *VEGF* in exudative AMD [83,84] and is an upstream stimulator of the PI3K/Akt pathway [85] through the inhibition of *GSK-3β* [86]. Moreover, β-catenin signaling inhibition is associated with the decrease of the PI3K/Akt pathway [87,88]. Numerous studies have shown that the PI3K/Akt pathway is activated in exudative AMD [67,68,69] and can stimulate both *HIF-1α* and *VEGF* [69]. VEGF production is stimulated by *HIF-1α* to deteriorate the functions of choroid and retinal endothelial cells and to stimulate angiogenesis in exudative AMD [70,71,72]. The activation of *LDH-A* is associated with *VEGF* stimulation [89,90,91,92]. Thus, the accumulation of lactate in the cytosol stimulates *VEGF* activity [93,94,95]. CNV formation is directly stimulated by overexpressing *VEGF* [96,97,98,99]. 

## 7. CRs and Aerobic Glycolysis 

**Figure 3:**
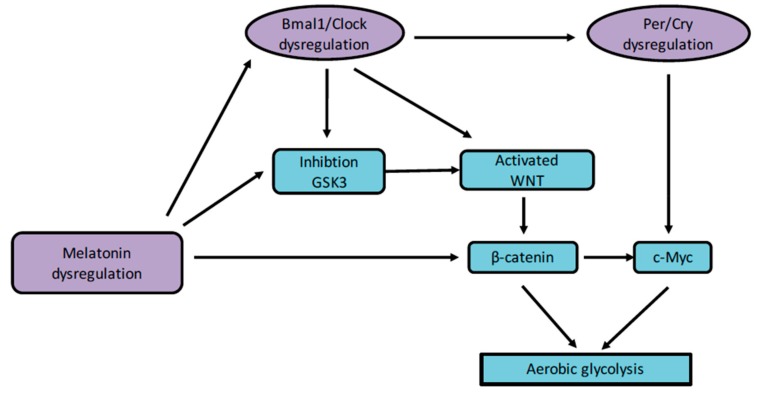
CRs and aerobic glycolysis. The dysregulation of melatonin and circadian clock genes leads to the activation of WNT and inactivation of *GSK3*. The WNT pathway enhances β-catenin accumulation and then, its nuclear translocation. Activation of the β-catenin pathway leads to aerobic glycolysis. Abbreviations: circadian locomotor output cycles kaput (*Clock*), brain and muscle aryl-hydrocarbon receptor nuclear translocator-like 1 (*Bmal1*), period (*Per*), cryptochrome (*Cry*), glycogen synthase kinase-3β (*GSK-3β*).

Few studies have focused on the relationship between CRs and aerobic glycolysis (Figure 3). Nevertheless, this relation could be mainly interesting in the development of tumors [100]. In the same way, melatonin modulation by CRs in cancers is associated with the disruption of aerobic glycolysis [101,102,103]. Thermodynamic and energy reprogramming highlight this relation in fibrosis [104], in neurodegenerative diseases [105,106], and in cancers [107]. The importance of 24-h fluctuations in aerobic glycolysis and the availability of NADPH in cancer have been shown through the consideration of the redox influence of NADPH [108]. 

## 8. The Canonical WNT/β-Catenin Pathway 

The Wingless/Int (WNT) pathway is a family of secreted lipid-modified glycoproteins [109]. Several pathophysiological processes are mediated by this pathway including fibrosis and angiogenesis [17,110,111].

**Figure 4:**
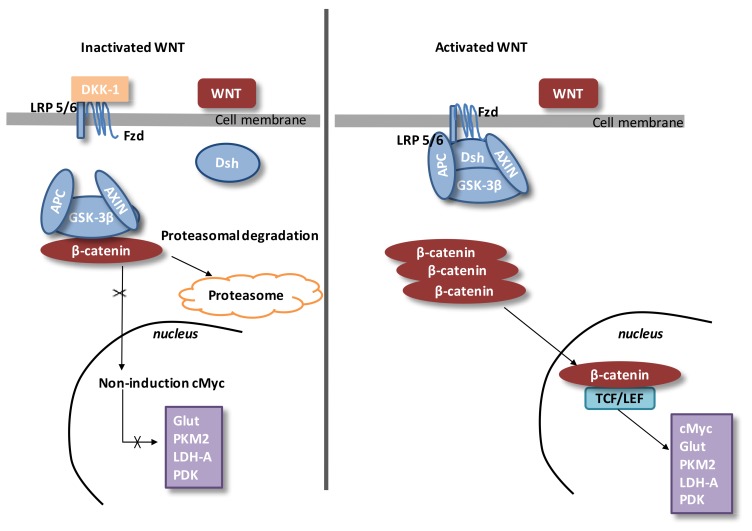
The canonical WNT/β-catenin pathway. Downregulated WNT. Under physiological conditions, the cytosolic β-catenin is bound to its complex destruction, which consists of *APC*, *AXIN,* and *GSK-3β*. *GSK-3β* phosphorylates β-catenin. Then, phosphorylated β-catenin is suppressed into the proteasome. The cytosolic β-catenin level is conserved low in the absence of WNT ligands. If β-catenin is not accumulated in the nucleus, the *TCF/LEF* complex does not activate its target genes. *DKK1* downregulates the WNT/β-catenin pathway through the link to WNT ligands or *LRP5/6*. Upregulated WNT. When WNT ligands stimulate *FZD* and *LRP5/6*, *DSH* is activated and phosphorylated by *FZD*. In turn, phosphorylated *DSH* stimulates *AXIN*, which comes off the β-catenin destruction complex. β-catenin escapes from phosphorylation to accumulate in the cytosol. Cytosolic β-catenin goes into the nucleus, where it complexes with *TCF/LEF* and activates the transcription of target genes. Abbreviations: tumor suppressor adenomatous polyposis coli (*APC*), circadian locomotor output cycles kaput (*Clock*), brain and muscle aryl-hydrocarbon receptor nuclear translocator-like 1 (*Bmal1*), dickkopf-related protein 1 (*DKK-1*), disheveled (*DSH*), period (*Per*), cryptochrome (*Cry*), glycogen synthase kinase-3β (*GSK-3β*), frizzled (*FZD*), lactate dehydrogenase A (*LDH-A*), low-density lipoprotein receptor-related protein 5/6 (LRP 5/6), glucose transporter (*Glut*), pyruvate dehydrogenase kinase (*PDK*), and t-cell factor/lymphoid enhancer-binding factor (*TCF/LEF*).

During eye development, WNT/β-catenin pathway activity is highly mediated. Then, a dysfunction of the WNT/β-catenin pathway leads to several ocular malformations due to defects in cell fate differentiation [112]. During the development of lens, the WNT/β-catenin pathway is stimulated in the periocular surface ectoderm and lens epithelium [113,114]. For retinal development, the WNT/β-catenin pathway is stimulated in the dorsal optic vesicle and then participates in the activation of retinal pigment epithelium (RPE) at the optic vesicle step. At this level, the WNT/β-catenin pathway is contained inside the peripheral RPE [115]. The retinal vascular initiation is mainly modulated by the expression of the WNT/β-catenin pathway [112]. In the retinal vascular system, the WNT/β-catenin pathway is controlled by the erythroblast transformation-specific (ETS) transcription factor *Erg*. *Erg* has a main role in angiogenesis [116]. *Erg* modulates the WNT/β-catenin pathway by promoting β-catenin stability and by regulating the transcription of Frizzled 4 (*FZD4*) (Figure 4) [116].

Stimulation of FZD4/β-catenin signaling needs the presence of the complex *LRP5/LRP6* [117]. *LRP5* has a main role while *LRP6* has a minor role in retinal vascularization [118,119]. Disheveled (Dsh) forms a complex with *AXIN*, and this prevents the phosphorylation of β-catenin by glycogen synthase kinase-3β (*GSK-3β*). Then, β-catenin accumulation in the cytosol is observed and translocates to the nucleus and binds the T-cell factor/lymphoid enhancer factor (*TCF/LEF*) co-transcription factors. This nuclear binding allows the transcription of WNT-responsive genes such as *cyclin D1, c-Myc, PDK1*, and *MCT-1* [120,121]. 

In the absence of WNT ligands, cytosolic β-catenin is phosphorylated by *GSK-3β*. 

A destruction complex is composed of tumor suppressor adenomatous polyposis coli (APC), *AXIN, GSK-3β*, and β-catenin. Then, phosphorylated β-catenin is destroyed into the proteasome. WNT inhibitors including *DKKs* and *SFRPs* control the WNT/β-catenin pathway by preventing its ligand–receptor interactions [122].

*GSK-β*, an intracellular serin-threonin kinase, is a regulator of the WNT pathway [123]. *GSK-3β* regulates numerous pathophysiological pathways (cell membrane signaling, neuronal polarity, and inflammation) [124,125,126]. *GSK-3β* downregulates β-catenin cytosolic accumulation and then its nuclear translocation [124]. *GSK-3β* diminishes β-catenin, the mTOR pathway, *HIF-1α*, and *VEGF* expression [76]. 

## 9. The Canonical WNT/β-Catenin Pathway in Exudative AMD

Aberrant activation of the WNT/β-catenin pathway is associated with focal retinal degeneration and exudative AMD lesions [127]. Kallistatin, an endogenous antagonist of the WNT/β-catenin pathway, is inhibited in AMD adults [127]. Kallistatin, a member of the serine proteinase inhibitor (*SERPIN*) family, can lead to anti-angiogenic and anti-inflammatory properties [78,128,129,130,131,132]. Kallistatin forms a complex with *LRP6* to inhibit the WNT/β-catenin pathway [131,132]. In murine models with focal retinal AMD-like lesions, administration of the anti-LRP6 antibody downregulates the WNT/β-catenin pathway and stops the formation of retinal lesions [127]. 

Tissue factor (*TF*), a transmembrane cell-surface receptor for plasma coagulation factor VII, is considered as the major effector of the extrinsic coagulation pathway [133]. TF possesses proangiogenic roles in the stages of neovascularization formation [134,135,136]. Activation of TF is associated with exudative AMD retina [135] and its activation allows the initiation of exudative AMD through inflammatory processes [135,137,138,139] and angiogenesis [139,140]. *TF* activates *VEGF* expression and participates in vascular formation by activating the WNT/β-catenin pathway [141]. The WNT/β-catenin pathway is downregulated in CNV models by the overexpression of Mab2F1 to reduce retinal vascular leakage [83,142]. Mab2F1 is a monoclonal antibody specific for *LRP6*, and its use shows the main role played by the WNT/β-catenin pathway in exudative AMD. 

A recent study has observed that the diminution of *DKK-1* circulating levels was correlated with exudative AMD [80]. Decreased levels of *DKK-1* are associated with exudative AMD severity and CNV initiation [80]. Causes of decreased *DKK-1* levels are not well-known, but some studies have shown that circulating *DKK-1* originates from the platelets [143].

The stimulation of the WNT/β-catenin pathway leads to the over-expression of *VEGF, TNF-α*, and *ICAM-1* [78,81,82]. *VEGF*-activated by *TNF-α* has a key role in CNV [96,97,98,99]. TNF-α is overexpressed in exudative AMD [144,145], whereas *ICAM-1* is still constitutively expressed in the RPE and plays a main role for leukocyte adherence [146,147]. Numerous studies have reported several relationships between the WNT/β-catenin pathway and inflammation including *TNF-α* and *NF-λB* [17,148,149]. Inflammation is a main factor in AMD through the stimulation of *VEGF* activated by the WNT/β-catenin pathway [78,79,150,151].

## 10. CRs and WNT/β-Catenin Pathway

RORs are upstream effectors of the WNT/β-catenin pathway [152]. Through this interaction, circadian genes can modulate the cell cycle progression [153]. A *Bmal1* knockdown can downregulate the WNT/β-catenin pathway [154]. In wild-type mice, the levels of WNT-related genes are higher than those observed in *Bmal1* knockdown mice [155,156]. The proliferation of cells is controlled by *Bmal1* through the activation of the WNT/β-catenin pathway [157]. *Bmal1* involves β-catenin transcription, diminishes β-catenin degradation, and inhibits *GSK-3β* activity [158]. In the intestinal mucosa of ApcMin/+ mice, the degradation of *Per2* leads to an increase in β-catenin through circadian disruption [159]. 

In normal conditions, the core circadian genes operate in accurate feedback loops and keep the molecular clockworks in the suprachiasmatic nucleus (SCN). They allow for the control of peripheral clocks [24,39]. *Per1* and *Per2* maintain cell CRs and modulate cell-related gene activity such as *c-Myc*, so as to sustain the physiological cell cycle [160,161]. 

## 11. Aerobic Glycolysis and the WNT/β-Catenin Pathway

**Figure 5:**
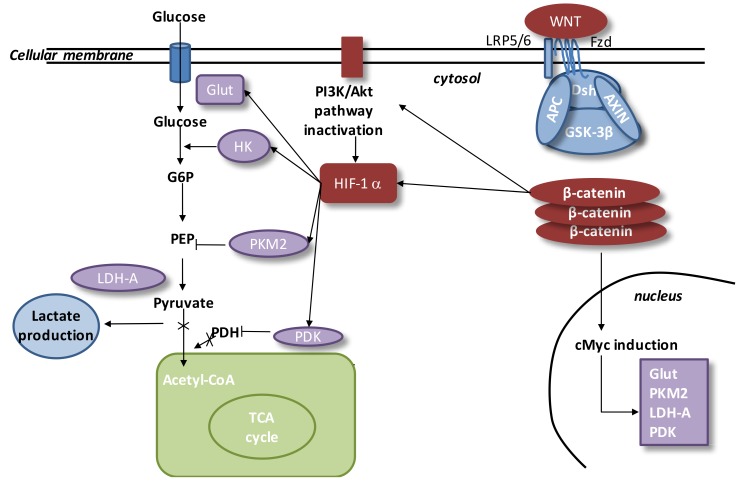
Interactions between the WNT pathway and aerobic glycolysis in exudative AMD. In exudative AMD, the WNT pathway is activated. In the presence of WNT ligands, cytosolic β-catenin is accumulated in cytosol and *GSK-3β* is inhibited. *APC* and *Axin* combine with *GSK-3β* and *DSH* to form a complex with *LRP 5/6* and *FZD*. β-catenin translocates to the nucleus and binds to *TCF/LEF* co-transcription factor. WNT target genes such as *cMyc* are activated. β-catenin accumulation increases the level of the PI3K/Akt pathway and results in activation of *HIF-1α*. Activated *HIF-1α* stimulates *Glut, HK, PKM2, LDH-A,* and *PDK1*. Activation of *HIF-1α* involves *PKM2* translocation to the nucleus. *PKM2* activates the PEP cascade and the formation of pyruvate. *PKM2* binds to β-catenin and induces cMyc-mediated expression of glycolytic enzymes (*Glut, LDH-A, PDK1*). Activation of *Glut* and *HK* involves glucose hyper-metabolism with an increase in glucose transport and phosphorylation rates. *PDK1* inhibits *PDH* and downregulates the pyruvate entrance into mitochondria. Lactate production is activated by *LDH-A*. This is the aerobic glycolysis. Abbreviations: tumor suppressor adenomatous polyposis coli (*APC*), dickkopf-related protein 1 (*DKK-1*), glucose 6 phosphate (*G6P*), disheveled (*DSH*), hexokinase (*HK*), glycogen synthase kinase-3β (*GSK-3β*), lactate dehydrogenase A (*LDH-A*), glucose transporter (*Glut*), phosphoenolpyruvate (*PEP*), pyruvate dehydrogenase kinase (*PDK*), t-cell factor/lymphoid enhancer-binding factor (*TCF/LEF*), frizzled (*FZD*), low-density lipoprotein receptor-related protein 5/6 (LRP 5/6), and tricarboxylic acid cycle (*TCA*).

Some reports have highlighted that the WNT/β-catenin pathway is a main effector of aerobic glycolysis (Figure 5) [10,15,20,66,104]. The PI3K/Akt pathway stimulates glucose metabolism to enhance protein and lipid syntheses [162]. Moreover, the PI3K/Akt pathway increases glucose metabolism to protect cells against reactive oxygen species (ROS) stress induced by activated *HIF-1α* [163]. HIF-1α stimulates pyruvate dehydrogenase kinase (*PDK*) to phosphorylate *PDH* and inactivates it, leading to cytosolic pyruvate being shunted into lactate by *LDH-A* [164]. *HIF-1α* is transcriptionally activated by the PI3K/Akt/mTOR pathway through 4E-BP1 and STAT3 [165,166,167,168,169,170]. 

Numerous studies have observed that the WNT/β-catenin pathway can downregulate pyruvate oxidation in the TCA cycle [20,171]. The WNT/β-catenin pathway, by activating both the PI3K/Akt/mTOR pathway and HIF-1α, can lead to aerobic glycolysis [171,172]. The PI3K/Akt pathway controls β-catenin accumulation [173]. *c-Myc* directly stimulates *HIF-1α* [174], *PDK*, and lactate transporter (*MCT-1*) expression [171]. The stimulation of HIF-1α leads to the overexpression of glucose transporters (Glut), hexokinase (*HK*), pyruvate kinase (*PK*), *PDK1*, and *LDH-A* [175,176,177,178]. 

## 12. Conclusions

Changes in energy metabolism are associated with metabolic and thermodynamic alterations and abnormal CRs in exudative AMD. In exudative AMD, the canonical WNT/β-catenin pathway is increased. Energy behaviors of metabolic enzymes in exudative AMD are modified by this upregulation of the WNT/β-catenin pathway, leading to the enhancement of aerobic glycolysis and thus the production of lactate. This explains the glucose hyper-metabolism observed in exudative AMD cells. The WNT pathway is driven by the CRs and operates under a circadian regime evolving to changes in energy metabolism. Regulation of the WNT/β-catenin pathway is directly controlled by CRs and the impairment of these rhythms is involved in reprogramming energy metabolism, enabling exudative AMD.

## Abbreviations

AMD	Aged-macular degeneration
Bmal1	Brain and muscle aryl-hydrocarbon receptor nuclear translocator-like 1
Clock	Circadian locomotor output cycles kaput
CRs	Circadian rhythms
Per	Period
RORs	Retinoid-related orphan receptors;
NF-κB	Nuclear factor κB
TNF	Tumor necrosis factor
